# Telomere elongation during early development is independent of environmental temperatures in Atlantic salmon

**DOI:** 10.1242/jeb.178616

**Published:** 2018-06-04

**Authors:** Darryl McLennan, John D. Armstrong, David C. Stewart, Simon Mckelvey, Winnie Boner, Pat Monaghan, Neil B. Metcalfe

**Affiliations:** 1 Institute of Biodiversity, Animal Health and Comparative Medicine, Graham Kerr Building, University of Glasgow, Glasgow, G12 8QQ, UK; 2 Marine Scotland–Science, Freshwater Laboratory, Faskally, Pitlochry, PH16 5LB, UK; 3 Cromarty Firth Fishery Trust, CKD Galbraith, Reay House, 17 Old Edinburgh Road, Inverness, IV2 3HF

**Keywords:** Oxidative stress, Environmental effect, Cell proliferation, Physiology, Fish, Telomeres

## Abstract

There is increasing evidence from endothermic vertebrates that telomeres, which cap the ends of chromosomes and play an important role in chromosome protection, decline in length during postnatal life and are a useful indicator of physiological state and expected lifespan. However, much less is currently known about telomere dynamics in ectothermic vertebrates, which are likely to differ from that of endotherms, at least in part due to the sensitivity of ectotherm physiology to environmental temperature. We report here on an experiment in which Atlantic salmon (*Salmo salar*) were reared through the embryonic and larval stages of development, and under differing temperatures, in order to examine the effects of environmental temperature during early life on telomere dynamics, oxidative DNA damage and cellular proliferation. Telomere length significantly increased between the embryonic and larval stages of development. Contrary to our expectations, variation in telomere length at the end of the larval stage was unrelated to either cell proliferation rate or the relative level of oxidative DNA damage, and did not vary between the temperature treatments. This study suggests that salmon are able to restore the length of their telomeres during early development, which may possibly help to buffer potentially harmful environmental effects experienced in early life.

## Abstract (precis)

**Summary:** The authors show that, in salmon, telomeres significantly lengthen between the embryonic and larval stages of development, and that this is not influenced by environmental temperature.

## INTRODUCTION

Conditions experienced in early life can have permanent effects on individual phenotypes (Lindström, 1999; Monaghan, 2008). Such effects might decrease or increase fitness, depending on whether the early life conditions were relatively poor or good. For example, environmental conditions during growth have been linked to future reproductive performance (Ohlsson et al., 2002; Spencer et al., 2003; Auer et al., 2010; Lee et al., 2012), adult body mass (Douhard et al., 2013) and competitive ability (Royle et al., 2005), as well as impacting long-term adult health (Gluckman et al., 2008). Notably, there has been increasing interest in the long-term effects of early life environmental conditions on ectotherms, since their development may be altered under the influence of an increasingly warming world (Zuo et al., 2012).

A potential mechanistic link between early life conditions and later life viability lies in the telomeres, which cap the ends of eukaryotic chromosomes and play an important role in chromosome protection (for reviews see Blackburn, 1991; Campisi et al., 2001; Monaghan, 2010). Telomere loss occurs at each round of cell division as a result of the ‘end replication problem’, but the amount of loss may in part be influenced by other factors, including levels of oxidative damage to the telomeric DNA (Reichert and Stier, 2017; Monaghan and Ozanne, 2018). Telomeric DNA has a high guanine content, which is particularly susceptible to oxidative damage, and there is relatively little repair of oxidative damage in the telomeric regions (Passos et al., 2007; Monaghan and Ozanne, 2018). A relatively short telomere length is generally considered indicative of relatively poor biological state. A growing number of studies have linked a shorter telomere length to reduced life expectancy and/or adult performance (Heidinger et al., 2012; Boonekamp et al., 2014; Cram et al., 2017; Salmón et al., 2017; Wilbourn et al., 2018), as well as to an increased probability of population extinction (Dupoué et al., 2017). Moreover, telomeres are known to undergo shortening in response to early life adversity (Herborn et al., 2014; Nettle et al., 2015; Ridout et al., 2017).

However, most of the telomere studies to date have focussed on humans (e.g. Broer et al., 2013) and other mammals and birds (Olsson et al., 2018), and these endothermic taxa mostly appear to downregulate telomere restoration mechanisms in post-embryonic somatic tissue and use replicative ageing as a tumour suppression mechanism (Gomes et al., 2010). In contrast, a number of studies have detected telomere restoration mechanisms (mostly by the enzyme telomerase) to extend into at least juvenile life in ectotherms (Yap et al., 2005; Peterson et al., 2015; Hatakeyama et al., 2016; Ujvari et al., 2017; Yip et al., 2017). Therefore, ectothermic species may be more likely to maintain their telomere length (and thus their genome stability) until later in life, compared with endotherms. This has presumably evolved in tandem with other life history traits and possibly alternative tumour protection mechanisms. It could also help to buffer the long-term consequences of potentially harmful environmental effects experienced in early life.

One of the most important factors that may influence ectotherm telomere dynamics is environmental temperature, with a number of studies linking shortened ectotherm telomeres to environmental temperatures that could be classified as stressful (Debes et al., 2016; Simide et al., 2016; Dupoué et al., 2017). However, non-stressful temperature variation could also affect early life telomere dynamics in ectotherms through several different routes. Firstly, ambient temperature directly affects the maintenance rate of metabolism (Jonsson and Jonsson, 2011) and so possibly also the production of reactive oxygen species (ROS) (Abele et al., 2002; Salin et al., 2015). It has been suggested that ROS act as the underlying mechanism that links variation in telomere length to environmental stressors (Haussmann and Marchetto, 2010), such as environmental temperature (Abele et al., 2002; Bae et al., 2016). However, more detailed studies are still needed to test this potential mechanism *in vivo* (Boonekamp et al., 2017; Reichert and Stier, 2017; Monaghan and Ozanne, 2018). It is interesting to note that, to date, two studies on fish did not find a significant correlation between temperature and levels of oxidative damage *in vivo* (Vinagre et al., 2012; Hemmer-Brepson et al., 2014).

Environmental temperatures also affect developmental rates. Individuals experiencing different temperature regimes could vary significantly in developmental stage for a given chronological age (and vice versa). Few studies have attempted to disentangle the separate effects of developmental rate and chronological age on telomere dynamics. However, it is possible that individual ectotherms that differ in developmental rate (but are of the same age) will also differ in telomere length, since developmental rate could be linked to rates of cell division and, potentially, levels of oxidative damage. For example, accelerated development has been associated with increased antioxidant levels in amphibian tadpoles, possibly to counteract an increased oxidative threat (Gomez-Mestre et al., 2013; Burraco et al., 2017). Alternatively, it is possible that telomere length could differ between individuals of different ages (but of the same developmental stage) since those that are relatively older at a given developmental stage may have accumulated more oxidative damage over time.

Finally, temperature can also affect the way that ectotherm embryos grow. Embryonic growth is due to a mix of both cell division (hyperplasia) and cell growth (hypertrophy), but the balance between the two processes depends on temperature (reviewed by Arendt, 2007). This has implications for telomere dynamics since telomere loss occurs during hyperplasia (i.e. at each cell division) but not during hypertrophy. Fish eggs incubated at warmer temperatures produce fry that have fewer but larger muscle fibres, indicating a shift in the balance from hyperplasia to hypertrophy (Stickland et al., 1988; Usher et al., 1994). A possible explanation for this effect is that levels of dissolved oxygen reduce with increasing water temperature, which will limit an individual's capacity for cell proliferation (Matschak et al., 1997). Therefore, embryos developing in relatively warmer water appear to be achieving growth by disproportionately increasing the size of their existing cells (hypertrophy), which might result in having longer telomeres for a given body size than conspecifics developing in colder water.

In this experiment, we examined telomere length at two early life stages (embryo and larva) in Atlantic salmon (*Salmo salar* Linnaeus 1758), and whether this was influenced by differences in environmental temperature. We also examined two potential mechanistic pathways (levels of oxidative DNA damage and rates of cell proliferation) that may link temperature and telomere length, with the predictions that: (A) there would be a positive relationship between incubation temperature and levels of oxidative damage, and (B) there would be a negative relationship between incubation temperature and cell proliferation rate, with a relative shift from cell division to cell growth at relatively warmer temperatures.

## MATERIALS AND METHODS

### Study species

Salmon have two distinct life stages prior to being independent of maternal yolk and feeding on exogenous food. Embryos spend the first several months of development encapsulated within an egg membrane, referred to as the ‘eyed embryo’ stage, when the dark eyes of the developing embryo are visible through the transparent membrane. Embryos generally develop eyes (i.e. reach the eyed stage) roughly half way between the dates of fertilisation and hatching (Verspoor et al., 2007). The embryo then hatches into the alevin (larval) stage, in which it gradually becomes more able to swim as the attached yolk sac is depleted. Complete resorption of the yolk sac marks the end of larval development and the transition to the first-feeding fry stage. Salmon are ideally suited to this study because they reproduce by external fertilisation [and so their matings can be easily controlled using *in vitro* fertilisation (IVF)]. They produce large clutches of relatively large eggs with a long embryonic and larval period (typically around 5–6 months between fertilisation and first feeding at average water temperatures), which can therefore be divided between temperature treatments, thereby enabling the genetic mix in different experimental groups to be controlled.

### *In vitro* fertilisation

In this experiment, we used a full-sibling IVF design, utilising parents that had spent only one winter at sea (1SW) before returning to the river to spawn. The time spent at sea was initially determined by size (1SW fish are usually smaller than those that remain at sea for longer) but subsequently confirmed by scalimetry (Shearer, 1992). On 8 December 2014, wild parent fish were captured at the Loch na Croic fish trap on the River Blackwater system, northern Scotland (57°60′N, 4°63′W), whilst undertaking their return spawning migration (see McLennan et al., 2016, 2018a, for further details of the standard capture procedure). Prior to the stripping of gametes, parent fish were anaesthetised using a 5% benzocaine solution. Each 1SW female was blotted dry and stripped of eggs, with approximately 150 eggs being retained for this experiment whilst the rest of the clutch was used for stocking purposes by the hatchery. A randomly chosen 1SW male was then blotted dry and stripped of sperm to fertilise the experimental sample of eggs. This design was repeated 22 times (using new fish each time) to produce 22 independent full-sib families, all fertilised on 8 December 2014. After mixing the sperm and eggs together for several minutes, each egg batch was washed and then placed in fresh water for at least 1 h to promote water hardening (the stage at which the eggs absorb water and become firm).

### Temperature treatment

The experimental eggs were then transferred to the aquarium facilities at the University of Glasgow, Scotland. Each of the 22 families of eggs was divided into three equal batches, with each batch being allocated to a temperature treatment group (4, 6 or 8°C). The lowest of these represented the ambient water temperature in the source river during the egg incubation period [December–February mean temperature: 3.90±0.91°C (mean±s.d.), McLennan et al. (2016), from the previous winter], whereas 6 and 8°C represented relatively warmer embryonic environments within the natural range experienced by this species (Jonsson and Jonsson, 2011). For each treatment group, eggs were held in baskets (one per family) distributed randomly within a tank (100×100×40 cm) that was oxygenated by a standard air stone. All treatment groups were held overnight at 4°C. The 6 and 8°C groups were then slowly increased from 4°C to their target temperature at a rate of 0.5°C day^−1^. Water temperatures within each of the treatment tanks were held at the required temperature using automated water chillers (Teco Tr25, Ravenna, Italy). Eggs were checked daily and any dead individuals were removed.

**Fig. 1.**
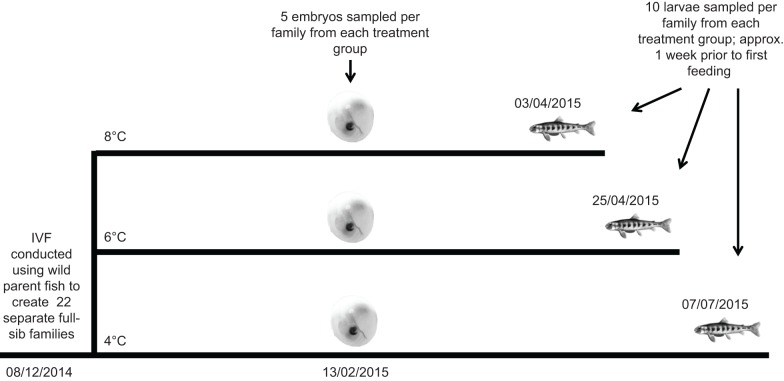
**Outline of experimental timeline.** All experimental crosses were conducted on 8 December 2014. All treatment groups were initially held overnight at 4°C, to represent ambient water temperature in the native stream. The temperature treatment then started on 9 December 2014, with temperatures in the 6 and 8°C groups slowly being increased from 4°C to their target temperature at a rate of 0.5°C day^−1^.

On 13 February 2015, by which time all families had reached the eyed embryo stage, five individuals were sampled per family (110 in total) from each of the three treatments, and were flash frozen for subsequent analysis of telomere length and DNA damage at the eyed embryo stage of development (hereafter referred to as the embryo stage). Sampled embryos were all the same chronological age (days since fertilisation) but at different developmental stages due to the temperature manipulation, with embryos in the warmest treatment having the most advanced development (Fig. 1).

The remaining embryos were allowed to continue development through to the end of the larval stage when the fish were close to taking exogenous food. This was identified by regularly checking the size of the remaining yolk sac until only a minimal amount remained, following the approach of Metcalfe and Thorpe (1992). When all families within a given treatment were estimated to be approximately 1 week prior to the exogenous food stage, again following the approach of Metcalfe and Thorpe (1992), ten larvae per family were randomly collected, killed, and measured for fork length and body mass. Five of the larvae were stored in 10% formaldehyde solution for subsequent immunohistochemical analysis (110 in total from each temperature treatment), and the remaining five from each family (110 in total from each temperature treatment) were flash frozen for subsequent analysis of late larval telomere length and DNA damage. The 8, 6 and 4°C treatment fish reached this late larval stage (hereafter referred to as the larva stage) on 3 April, 25 April and 7 July 2015, respectively. As a result, these larva samples were all at the same developmental stage but differed in chronological age (due to the temperature manipulation, see Fig. 1). For additional confirmation that we had sampled the larvae in each of the temperature treatments at approximately the same developmental stage, we calculated the mean coefficient of variation for body mass and body length across the three temperature treatments (which were 2.96 for mass and 1.48 for length). In addition, we also assessed how similar the heterogeneity of body mass and length was across the three temperature treatments by calculating the coefficient of variation (CV) across families in a given treatment. For body mass, the CV was 11.9, 11.1 and 12.3 at 4, 6 and 8°C, respectively, whereas, for body length it was 2.5, 3.1 and 2.5 at 4, 6 and 8°C, respectively. This suggests that no selective disappearance of slower- or faster-growing individuals had occurred. The larva samples that were fixed in 10% formaldehyde were transferred to 70% ethanol 3 days after sampling, for long-term storage at room temperature. Mortality was minimal (we estimate <5%) and was similar across all of the families and temperature treatments, again suggesting that there was little likelihood of any bias arising from selective disappearance. However, for reasons unknown, all larvae from family 8 that were incubated at the intermediate 6°C temperature treatment experienced mass mortality over the course of a few days. Therefore, there were 21 samples for the larva stage at this temperature treatment.

### DNA extraction and telomere analysis

DNA extraction and telomere analysis were conducted on pooled tissue samples (either 5 embryos or 5 larvae) for each family at each of the temperature treatments. The flash frozen embryos and larvae from each family were thawed slowly in ice-cold phosphate-buffered saline (PBS). Embryos were homogenised using a disposable tissue grinder, whereas larvae were homogenised in 1 ml of ice-cold 1×PBS in a 2 ml tube containing a ceramic bead. The homogenates were then passed through a 100 µm mesh cell strainer along with ice-cold PBS to a total volume of 50 ml. Samples were left for 1 min to allow larger aggregates of tissue to settle at the bottom of the flask. For each sample, 3 ml of supernatant (containing the single-cell suspension) was then transferred to a clean 15 ml centrifuge tube and centrifuged at 500 ***g*** for 10 min at 4°C. The supernatant was then discarded and cell pellets were held on ice until ready for extraction.

DNA extraction was performed on the cell pellets to which 180 µl buffer ATL+20 µl of proteinase K solution (20 mg ml^−1^) was added. Samples were then incubated at 56°C until cells were fully lysed. DNA was then extracted from the lysate using the DNeasy Blood and Tissue Kit (Qiagen, Hilden, Germany), following the manufacturer's protocol. Each set of DNA extractions conducted also included a negative control, which contained all of the reagents but without any tissue. This was used to check for contamination during the lysis and extraction steps. DNA concentration and purity was measured spectrophotometrically using a Nanodrop 8000 spectrophotometer (ThermoFisher Scientific, Waltham, MA, USA).

Telomere length was measured in all samples using quantitative PCR (Cawthon, 2002), and data were analysed using qBASE software for Windows (Hellemans et al., 2007), as described in McLennan et al. (2016). Interstitial telomere sites (ITS) could potentially add noise to the qPCR measurement; however, Atlantic salmon chromosomes are not thought to have a significant amount of ITS (Perez et al., 1999). In brief, the qPCR method produces a relative measurement of telomere length (RTL) and is calculated as a ratio (T:S) of telomere repeat copy number (T) to a stable copy number gene (S). In this experiment, we used the well-established glyceraldehyde-3-phosphate dehydrogenase (*GAPDH*) gene as the S gene. Performing a BLAST search on the Genbank sequence used in our *GAPDH* primer design (accession number: NM_001123561) confirmed that this sequence is present at a single location in the Atlantic salmon genome. There was also little variability in the *C*_t_ values for the *GADPH* PCR, which indicates a stable frequency in the genome. In addition to the samples, each plate also included a sixfold serial dilution of a reference sample (1.25–40 ng well^–1^) and a non-target control (NTC). The DNA for the serial dilution was a pool of 24 samples that included both life stages (embryo and larva). The serial dilution was used to generate a standard curve and calculate assay efficiencies. The NTC contained all reaction components apart from DNA and was included on each plate (in triplicate) to check for non-specific binding and potential contamination between sample wells. The mean assay efficiencies for the telomere and GAPDH were 91.9 and 94.5%, respectively, well within the acceptable range (85–115%). The average intraplate variation of the *C*_t_ values was 1.43 for the telomere assay and 0.49 for the GAPDH assay, respectively. The average interplate variation of the *C*_t_ values was 1.88 for the telomere assay and 1.10 for the GAPDH assay, respectively. The six points of the standard curve were used to calculate the inter-assay coefficient of variability of the telomere measurements (which was 9.33).

### Oxidative DNA damage assay

Oxidative damage to DNA was quantified by measuring levels of 8-hydroxy-2′-deoxyguanosine (8-OHdG). 8-OHdG is a product of the DNA excision-repair mechanism that occurs when the base guanine is oxidatively damaged; therefore, it is generally accepted as a sensitive marker of the oxidative challenge to DNA at the time of measurement (Valavanidis et al., 2009). The same extract of DNA was used as for the telomere analysis. We quantified 8-OHdG using a commercial kit, following the manufacturer's protocol (Epigentek, Farmingdale, NY, USA, item no. P-6003). In brief, the colourimetric kit detected 8-OHdG by using capture and detection antibodies specific to 8-OHdG. From this, a relative quantification of 8-OHdG was calculated as the percentage of DNA in a sample that was damaged. Each plate included a five-point standard curve that was made from a positive control of known damage. The assessment of damage in the unknown samples was adjusted according to the absorbance readings of the standard curve, so that potential inter-plate variation was partially accounted for. The level of DNA damage in our samples was relatively low; therefore, we used a more dilute standard curve (1.56–25 pg µl^−1^ 8-OHdG) than that suggested in the manufacturer's protocol (5–200 pg µl^−1^ 8-OHdG). The two life stages of a given family (i.e. embryo and larva) were measured on the same plate. All samples were run in duplicate, and the replicate measurements were found to be consistent (intra-class correlation coefficient=0.821; *F*_131,131_=5.90, *P*<0.001).

### Muscle cell proliferation analysis

For this experiment, we chose to quantify the rate of cell proliferation in the anterior muscle of the larvae, since muscle is by far the largest tissue in Atlantic salmon and somatic growth is closely linked to variation in muscle cell proliferation (Weatherley et al., 1988). To do so, we conducted an immunohistochemical analysis using an antibody stain specific to the proliferative cell nuclear antigen (PCNA) protein. PCNA is essential in cell replication and is actively expressed in nuclei during the DNA synthesis phase of cell division (Yu et al., 1992). It has been successfully used to measure cell proliferation rates in a variety of fish species (for examples, see Ortego et al., 1994; Kilemade et al., 2002; Abdo et al., 2014; Peterson et al., 2015).

Immunohistochemistry was performed by the University of Glasgow Veterinary Diagnostic Service, Scotland. For each treatment, two individual larvae per family (that had been preserved in formaldehyde and stored in ethanol) were embedded longitudinally in paraffin and sectioned at a thickness of 2.5 µm. Antigen retrieval (i.e. the exposure of antigenic sites, allowing antibodies to bind) was employed using heat-induced epitope retrieval (HIER) in a sodium citrate buffer, pH 6, for 10 min at 110°C. Sections were stained using a Dako autostainer. The primary antibody was PCNA [Santa Cruz PCNA (FL-261) cat. no. sc-7907], dilution 1:100, incubated for 30 min. The secondary antibody (which binds to the primary antibody and assists in detection) was Dako EnVision+System HRP-labelled polymer anti-rabbit, incubated for 30 min at room temperature. Visualisation was achieved using Dako+ 3,3′-diaminobenzidine (DAB) substrate and chromogen. Sections were counterstained with the nuclei-specific Gills Haematoxylin for 27 s.

**Fig. 2.**
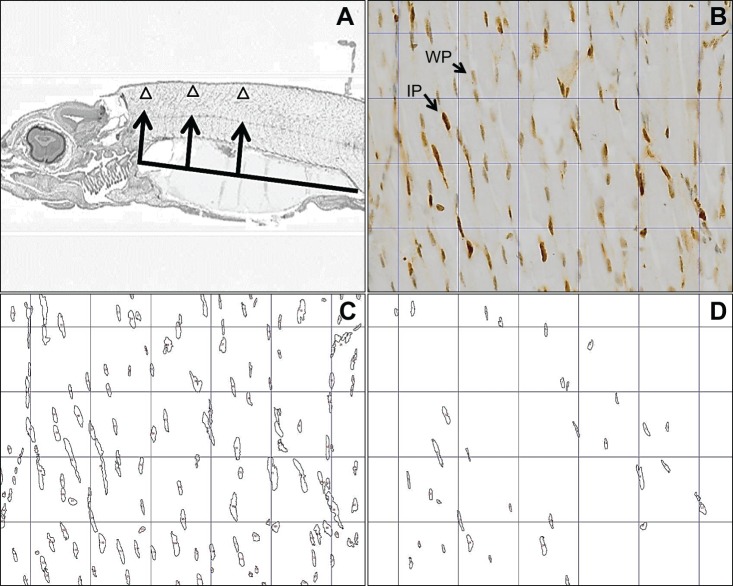
**Outline of proliferative cell nuclear antigen** (**PCNA) cell proliferation analysis.** (A) Example of a scanned immunohistochemical slide, showing how the three regions of larval dorsal myotome muscle were selected and used for 26× image capture. The horizontal thick line is the maximum longitudinal axis of the remains of the yolk sac. The triangles demonstrate the three most dorsal regions in relation to the anterior-most point, the 1st quartile and the 2nd quartile point of the horizontal line. Triangles are also representative of the size of the area that was used for image capture. (B) Example of an original image showing intensely PCNA-positive nuclei (IP) and weakly PCNA-positive nuclei (WP). (C) The same image filtered using ImageJ software to select all nuclei (IP and WP). (D) The same image filtered to now show only the IP nuclei. The percentage of cells actively undergoing replication was calculated as 100×(IP/all nuclei).

Stained slides were then scanned at the Marine Scotland Marine Laboratory in Aberdeen, using the Dot Slide virtual microscopy system (version 2.5) at a resolution of 40×. In order to select the same body regions for analysis on all specimens, a reference line was drawn along the maximum longitudinal axis of the remaining yolk sac of each larva and the anterior-most point, the 1st quartile and the 2nd quartile point along this line were identified. The most dorsal region of myotome muscle was identified in relation to these three points and used for image capture (Fig. 2A). Images were captured at 26× magnification (area=0.03 mm^2^). Since there were two larvae from the same family per temperature treatment, and three images were captured per larva, this gave six images per family from each treatment.

Previous studies have shown that PCNA has a relatively long half-life, meaning that residual PCNA may continue to be present in cells that have fully completed division (Scott et al., 1991). In addition, there is a relatively high cell turnover rate at the embryonic stage, which meant that most cells had incorporated some of the PCNA-specific stain. To overcome the noise of this historical cell division, we adopted the approach of Kilemade et al. (2002) in distinguishing between intensely PCNA-positive (IP) nuclei (i.e. those actively dividing) and weakly PCNA-positive (WP) nuclei (those no longer dividing), using ImageJ to distinguish objectively and consistently which nuclei were intensely PCNA positive.

We first quantified the total number of nuclei in an image by conducting the following three steps: (1) the image was converted into an 8 bit greyscale format; (2) stained areas were identified by selecting all pixels that had an intensity between 0 and 165 (0=black, 255=white); and (3) the Analyse Particle tool in ImageJ was then used to differentiate between true nuclei (which we set as a minimum of 120 clustered pixels) and smaller randomly stained artefacts (see Fig. 2C for an example of the output from this process). The number of nuclei was then counted by ImageJ. We then used ImageJ again to analyse the same image, but this time to identify only nuclei that were intensely PCNA positive (IP), by repeating the above three steps but this time increasing the 8 bit intensity threshold to <90 rather than <165 (Fig. 2D). Therefore, by using ImageJ to separately identify the total number of nuclei (Fig. 2C) and the number of IP nuclei (Fig. 2D) from the same image, we were able to estimate the percentage of nuclei in that image that were actively undergoing cell replication at the time of sampling. Furthermore, since all images were captured at the same magnification (and were therefore of the same area of tissue), we were also able to generate a cell size index by taking the inverse of the total number of nuclei per image (i.e. the higher the value, the larger the cells).

### Statistical analysis

In total, there were 22 telomere length and DNA damage measurements (one per family) for each of the three temperature treatments at both the embryo and larva stage, giving 132 data points for the telomere and DNA damage analyses. Cell proliferation was quantified at the larva stage only, giving 66 data points (one mean value calculated per family for each treatment). We measured/calculated the following variables to be used in statistical models, where appropriate: relative telomere length at the embryo (embryo RTL) and larva stage (larva RTL); the percentage of DNA that was damaged (i.e. % 8-OHdG) at the embryo (embryo DNA damage) and larva (larva DNA damage) stage; average fresh mass of the larvae, calculated as an average for each family (larva mass); the percentage of intensely stained PCNA nuclei at the larva stage (larva cell proliferation); the inverse of the number of cells per unit area, as a proxy for cell size (larva cell size index); the life stage at which a sample was taken (i.e. whether at the embryo or larva stage; subsequently referred to as life stage); and the aquarium temperature treatment (temp. treatment).

Firstly, we ran a linear mixed model (LME) to assess whether there was a significant change in telomere length between the two life stages and between the temperature treatments (model A). We then ran six further LMEs to assess factors affecting variation in larva mass (model B), larva cell proliferation rate (model C), embryo RTL (model D), larva RTL (model E), embryo DNA damage (model F) and larva DNA damage (model G). Family ID was included as a random factor in each analysis, to give a repeated measure design. For a full outline of the statistical models and the variables included in each, please see Table 1.

**Table 1.**
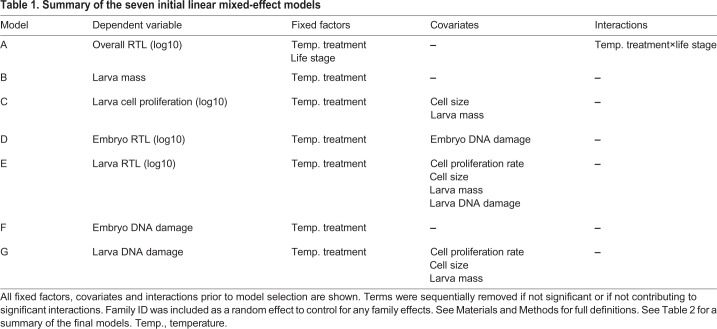
**Summary of the seven initial linear mixed-effect models**

We used a Pearson correlation coefficient matrix to assess potential collinearity between explanatory variables (with a cut-off coefficient of 0.7). Larva mass and length were identified as being highly collinear (Pearson *r*=0.81, *P*<0.001) and therefore only larva mass was used for subsequent analyses. The models were simplified using backwards model selection; the Akaike information criterion (AIC) was used during model fitting and variables were only removed from a model if this resulted in a relative reduction of the AIC score. All statistical analyses were carried out using IBM SPSS 22 for Windows. For a summary of the seven final linear mixed-effect models, please see Table 2.

**Table 2.**
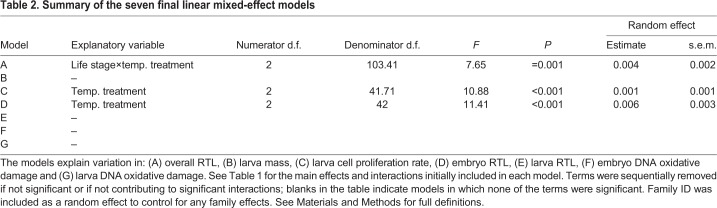
**Summary of the seven final linear mixed-effect models**

This experiment was approved by the University of Glasgow, Scotland local ethical review panel, and all procedures were carried out under the jurisdiction of a UK Home Office project licence (PPL 60/4292).

## RESULTS

### Telomere dynamics

**Fig. 3.**
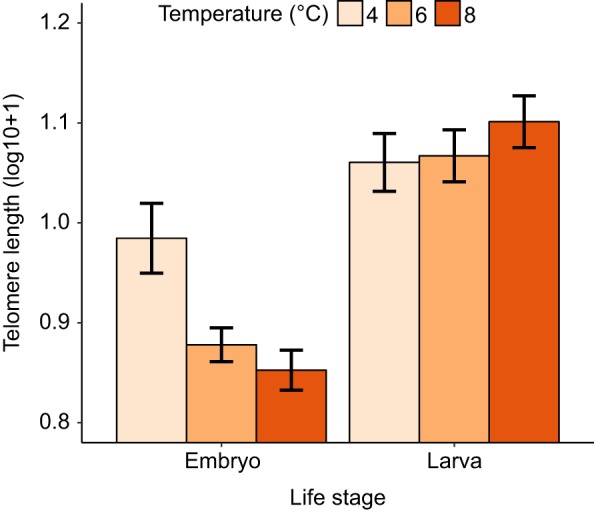
**The relative telomere length of the salmon at the embryo and larva stage in relation to incubation temperature.** Data are plotted as means±s.e.m., with each of 22 families contributing one data point per temperature treatment per life stage.

Telomere length significantly increased between the two life stages (embryo to larva) for each of the three temperature treatment groups. However, the relative amount of telomere elongation was dependent on the temperature treatment (significant life stage×temperature treatment interaction; Table 2A). Individuals reared at the coldest temperature had the longest telomere length when measured at the embryo stage (Table 2D, Fig. 3) but then underwent the smallest change in telomere length, so that, by the late larva stage, the three temperature treatment groups had similar telomere lengths (Fig. 3; note no effect of temperature treatment in Table 2E). However, these effects must be interpreted with caution since, at the embryo stage, all the sampling was done on the same day so embryos from the three temperature treatments were the same chronological age, but were at different stages of development (warmer temperatures=more advanced). In contrast, at the larva stage, all groups were at the same developmental stage (=completion of yolk absorption), but were of different chronological ages. Temperature treatment had no effect on the body mass of the larvae at the first feeding stage (Table 2B).

### Muscle cell proliferation rate

**Fig. 4.**
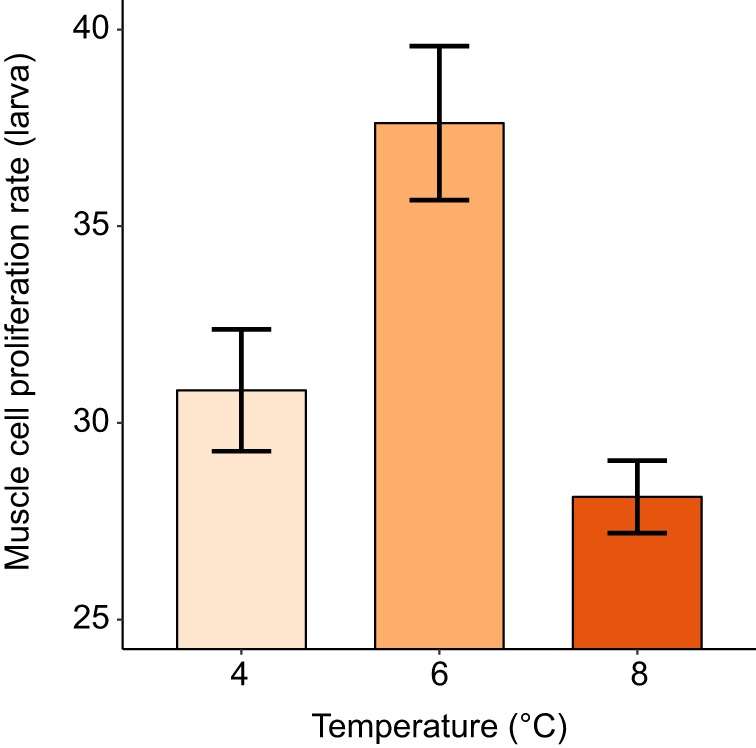
**The relationship between incubation temperature and the rate of muscle cell proliferation (i.e. the percentage of intensely-stained positive PCNA nuclei) at the late larval stage.** Data are plotted as means±s.e.m., with each of 22 families contributing one data point per temperature treatment.

The rate of cell proliferation in the anterior muscle of the larvae differed according to rearing temperature (Table 2C). Individuals in the intermediate temperature group had the highest percentage of intensely stained PCNA nuclei, i.e. the highest rate of cell proliferation (Fig. 4). However, this variation in cell proliferation rate was not significantly associated with the index of cell size, larva mass (Table 2C) or larva telomere length (Table 2E). The index of cell size was unrelated to telomere length.

### Oxidative DNA damage

Levels of DNA damage (8-OHdG) were not significantly different between the temperature treatments at either the embryonic (Table 2F) or the larval stage (Table 2G), nor was variation in DNA damage levels associated with telomere length at either life stage (Table 2D,E). It should also be noted that the level of DNA damage at each life stage was relatively low [5.2±0.5 pg (mean±s.e.m.) at the embryo stage and 8.3±0.7 pg at the larva stage), so that the majority of samples had damage levels at the lower end of the assay's detection limit (2 pg).

## DISCUSSION

In this study, we found that telomere length significantly increased between the embryonic and larval phases of development in Atlantic salmon. In a previous salmon study (McLennan et al., 2016), we also found a significant increase in telomere length between two time points at the embryonic stage of development, separated by 18 days. This study supports that finding, and further suggests that this telomere elongation extends beyond the embryonic stage and continues at least into the larval stage.

We found a negative correlation between developmental rate and telomere length at the eyed egg stage. All embryos were sampled on the same day, and were therefore all the same chronological age, but at slightly different developmental stages. Embryos reared at the coldest temperature (i.e. the least developed at the time of sampling, having only just reached the eyed stage) had the longest mean telomere length, whereas those reared at the warmest temperature (the most developed, having reached the eyed stage slightly earlier) had the shortest telomeres. It may be that telomeres decrease in length during embryonic development (e.g. because of rapid cell proliferation), but are then restored during later larval stage, perhaps due to temporal changes in telomerase activity (Hatakeyama et al., 2016). In support of this, there was no significant difference in telomere length between the three temperature treatment groups at the late larval stage, when all groups were at the same developmental stage but different chronological ages. This study used a cross-sectional approach because it is not currently possible to sample embryonic tissues non-destructively, which would be necessary for a longitudinal approach. As such, while it is possible that our finding of relatively longer telomeres at the larval stage could have been due to the selective mortality of embryos with shorter telomeres, this is extremely unlikely since mortality was minimal (we estimate <5%) and similar across all of the families.

In general, telomeres have been found to shorten with age, at least in many post-embryonic mammal and birds studied to date (Haussmann et al., 2003; Henriques and Ferreira, 2012), and it has been suggested that telomerase expression during human embryonic development is to maintain, rather than elongate, embryo telomere length (Liu et al., 2007). Much less is known about telomere dynamics during fish embryo development. We have shown in this study that telomere lengths in Atlantic salmon are significantly longer at the larval stage (i.e. at the point when the fish have used up all yolk reserves and begin to feed on exogenous food) compared with the earlier embryonic stage. Other studies of ectotherms have also found post-embryonic life stages in which telomeres appear to lengthen, even if they then decrease in late adulthood (Hatakeyama et al., 2016; Ujvari et al., 2017). Therefore, it may be that, in contrast to endotherms, telomerase expression in ectotherms in the later stages of development is capable of not only maintaining telomere length, but also elongating it, or that a different mechanism for restoring telomeres is at play, such as alternative lengthening of telomeres (ALT) (Henson et al., 2002). The reasons for this are currently unknown. However, it may be linked to the fact that most ectotherms are capable of growing through life, and so invest in telomere elongation during development in order to prepare cells for sustained proliferative capacity later in life (Gomes et al., 2010). Our results also suggest that salmon embryos may be able to regulate the length of their telomeres towards the end of larval development, thus compensating for loss at earlier embryonic stages.

The temperature manipulation was not linked to levels of oxidative damage to DNA at either the embryo or larva stage, nor was variation in DNA damage levels associated with variation in telomere lengths at either stage. There is still a limited understanding of how oxidative damage may affect telomere dynamics *in vivo* (Boonekamp et al., 2017; Reichert and Stier, 2017), and therefore it is possible that there is no mechanistic link between oxidative DNA damage and early life telomere dynamics in Atlantic salmon. However, there are also several points from this study that are worthy of discussion. Firstly, the level of DNA damage at each life stage was relatively low; therefore, these early life stages may not have accumulated sufficient oxidative damage to allow detection of significant patterns. It is also worth noting that we measured DNA damage by quantifying 8-OHdG, which is a product of the DNA excision–repair mechanism that occurs when guanine is oxidatively damaged, rather than being a direct marker of the DNA damage itself. Although the two are often highly correlated, it is also possible that the relatively low levels of 8-OHdG in our samples reflect reduced DNA repair activity at the time of measurement, irrespective of the levels of accumulated damage. It may also be that any potential temperature effects on oxidative damage were buffered by an upregulation of antioxidants, as suggested by Hemmer-Brepson et al. (2014). Another possibility is that any possible link between levels of DNA damage and telomere lengths was buffered by the significant telomere elongation we observed.

The rate of cell proliferation in the larvae differed significantly between the different temperature treatments, with salmon larvae that had been reared at the intermediate water temperature (6°C) having the highest cell proliferation rate. The optimal temperature for growth in juvenile Atlantic salmon is thought to be around 16°C (Elliott and Hurley, 1997). However, cell proliferation in Atlantic salmon is greatest at temperatures lower than the optimal growth temperature (Stickland et al., 1988; Usher et al., 1994; MacQueen et al., 2008). Considering these studies collectively, they suggest that cell proliferation rates may be maximised around 5°C in Atlantic salmon, which supports the pattern observed in this study. It is reasonable to assume that this temperature is warm enough to allow sufficient metabolism and growth to occur, yet cold enough that there will be a sufficient concentration of dissolved oxygen to allow this growth to occur by cell proliferation, since cell proliferation is oxygen demanding (Matschak et al., 1997).

Although incubation temperature influenced the rate of cell proliferation in the anterior muscle of the larvae, this was not linked to differences in mean telomere length. Again, it may be that the significant elongation of telomere length occurring in late larval development was buffering the effects of cell division on telomere lengths. It is not known how these relationships change in later life, when telomerase expression may be downregulated and growth and behaviour may have caused greater among-individual variation in physiological stress, oxidative stress levels and telomere dynamics. This is especially relevant in fish, where muscle fibre recruitment continues well beyond the embryo stage (Weatherley et al., 1988); indeed, active cell proliferation in muscle tissue is still detectable in late juvenile Atlantic salmon that have migrated to sea (Johnston et al., 2003).

We have shown that telomere length significantly increases during early development in Atlantic salmon, suggesting that ectotherms regulate early-life telomere dynamics in ways that contrast to patterns seen in endotherms. This supports observations made in other fish species (e.g. Hatakeyama et al., 2016). Our temperature treatment affected the development rate of the early-life salmon, as well as the rate of their cell proliferation. However, there was no significant difference in telomere length by the time the larvae were approaching the first feeding stage, suggesting that they are able to restore the length of their telomeres irrespective of the environmental temperature experienced during early development. These results contribute towards the assessment of how ectothermic species may alter their early-life physiology in order to acclimatise to changing environmental conditions. It would now be interesting to examine the longer-term effects of early-life environmental conditions on ectothermic telomere dynamics, as well as the potential associated telomere elongation mechanisms.

## Data Availability

Data are available from the Dryad Digital Repository (McLennan et al., 2018b): https://doi.org/10.5061/dryad.ct7h4ds
